# Dua Ti Dawa Ti*:* understanding psychological distress in the ten districts of the Kashmir Valley and community mental health service needs

**DOI:** 10.1186/s13031-019-0243-8

**Published:** 2019-12-12

**Authors:** Tambri Housen, Shabnum Ara, Akmal Shah, Showkat Shah, Annick Lenglet, Giovanni Pintaldi

**Affiliations:** 1 National Centre for Epidemiology and Population Health, Australian National University, ACT, Canberra, 2600 Australia; 2 Department of Psychology, University of Kashmir, Hazratbal, Srinagar, Jammu and Kashmir, 190006 India; 3 Ranchi Institute of Neuro-Psychiatry and Allied Sciences (RINPAS), Kanke Rd, Kanke, Ranchi, Jharkhand 834006 India; 4 Radbound University Medical Center, Geert Grooteplein Zuid 10, Nijmegen, 6525 GA Netherlands; 5 Médecins Sans Frontières, Plantage Middenlaan 14, 1018 Amsterdam, DD Netherlands

**Keywords:** Psychological distress, Mental health, Health service needs, Community perspective, Access, Exploratory methodologies, Qualitative, Kashmir

## Abstract

**Background:**

An extensive body of research exists looking at the level of psychological distress in populations affected by political conflict. Recommended response to psychological distress in humanitarian crises is still based on frameworks for interventions developed in western/European contexts including psychological first aid, counselling and group therapy. While there is growing, but limited, evidence that culturally modified interventions can lead to reduction in symptoms of psychological distress in conflict affected populations, there is a need to understand mental health help-seeking behaviour and mental health service needs from the perspective of affected communities.

**Methods:**

This study employed a qualitative exploratory research design based on principles of grounded theory. A combination of convenience and snowball sampling was used to recruit 186 adults from the general population to 20 focus group discussions; 95 men, median age 40 years, interquartile range (IQR): 27–48 years and 91 women, median age 40 years IQR: 32–50 years. Trained Kashmiri facilitators used a semi-structured interview guide to ascertain community perceptions on mental illness, help-seeking and service needs from the perspective of communities in the Kashmir Valley. Content analysis of transcripts resulted in the identification of seven overarching themes.

**Results:**

Common locally recognized symptoms of psychological distress were synonymous with symptoms listed in the Hopkins Symptoms Checklist (HSCL-25) and the Harvard Trauma Questionnaire (HTQ). Protracted political insecurity was highlighted as a major perceived cause of psychological distress in communities. Mental health help-seeking included traditional/spiritual healers in combination with practitioners of western medicine, with access highlighted as the main barrier. Divergent views were expressed on the effectiveness of treatment received. Participants’ expressed the need for investment in mental health literacy to improve the community’s capacity to recognize and support those suffering from psychological distress.

**Conclusions:**

Our findings demonstrate the universality of symptoms of psychological distress whilst simultaneously highlighting the importance of recognizing the cultural, spiritual and contextual framework within which psychological distress is understood and manifest. Co-constructed models of community based mental health services are needed.

## Background

Protracted conflict provides periods of peace interspersed with periods of political violence and insecurity. A recognized protective factor for improving mental health outcomes in populations affected by political conflict is the re-establishment of safety and security in the immediate environment [1, 2]. Likewise, persistent conditions of actual or perceived threat have been found to support the genesis and maintenance of psychological distress in affected populations [1, 3]. We conducted a qualitative study as part of ongoing activities to better understand the burden of psychological distress in the Kashmir Valley and specifically explore community perceptions of and reactions towards symptoms of psychological distress, document current health seeking behaviours and identify perceived mental health service needs.

Following the partition of India in 1947, the Kashmir Valley has been subject to continual political insecurity and ongoing conflict [4]. In 1989, an insurgency began leading to the displacement of over 100, 000 Kashmiri Pandits and 30 years of militant and military activity [5]. By 2015, approximately 70,000 Kashmiris had lost their lives in the conflict and 8000 people had been reported missing [6]. Frequent confrontations with violence have been reported including displacement, exposure to crossfire, ballistic trauma, round-up raids, torture, rape, forced labour, arrests/kidnappings and disappearances [7, 8]. The Office of the United Nations High Commission for Human Rights (OHCHR) 2018 report states that an estimated 130–145 civilians were killed by security forces between July 2016 and March 2018 and a further 16–20 killed by armed groups during the same period [9]. The loss of human life, human rights abuses and a resulting context of ongoing low-grade conflict has had its impact on Kashmir’s population. In addition, the effect of prolonged exposure to violence on the psychological well-being of the population has been confounded by natural disasters such as a 7.6 Mw magnitude earthquake in 2005 and floods in 2014. In Kashmir additional confounders include widespread poverty, uncertainty, grief, oppression and fear in addition to high unemployment with limited development of employment generating sectors.

The continuation of violent flare-ups in the Kashmir Valley leave many people in chronic heightened levels of stress, known locally as ‘*tension*’ or ‘*pareshani*’ due to the ‘*situation*’ or ‘*halaat*’. In 2015 Médecins Sans Frontières (MSF) partnered with the Department of Psychology at Kashmir University and the Institute of Mental Health and Neurosciences (IMHANS- Kashmir’s only psychiatric hospital), Kashmir’s only psychiatric hospital, to conduct a population based survey to provide baseline prevalence estimates of psychological distress in all ten districts of the Kashmir Valley [10, 11]. Prevalence estimates of probable depression, anxiety and PTSD in the Kashmir Valley were 41, 26 and 19%, respectively [11]. These estimates were based on scores on the 25 item Hopkins Symptoms Checklist (HSCL-25) for symptoms of depression and anxiety and the Harvard Trauma Questionnaire 16-item scale (HTQ-16) for Post-traumatic Stress Disorder. These tools are not diagnostic tools, diagnosis can only be confirmed via clinical interview with a clinical psychologist/psychiatrist, however, the high proportion of the adult population scoring above established cut-off scores demonstrated high levels of psychological distress in the Kashmiri population. Among the traumatogenic events witnessed or experienced, 47% had witnessed the violent death of someone they knew. Alarmingly, 12% of survey respondents reported having had thoughts of ending their life in the four weeks prior to the survey [12]. A rise in outpatient presentations for mental health issues has been reported by IMHANS from an average of 100 per week in 1980 to between 200 and 300 per day in 2013 [13]. In addition, the number of suicide attempts increased by more than 250% between 1994 and 2012 [14]. Mental health studies conducted in the Kashmir Valley consistently report a high prevalence of traumatogenic experiences and associated symptoms of psychological distress. Khan (2013) measured mental health outcomes in 390 probability sampled urban households in four administrative regions of Srinagar, reporting that 46% of the sample suffered from anxiety and 32%, depression [15]. Between 2003 and 2005, Margoob et al. [16] used clinical interviews conducted by psychiatrists to assess the prevalence of PTSD in 2391 probability sampled individuals from six districts of the Kashmir Valley. Prevalence of PTSD was found to be 7% with a life-time prevalence rate of PTSD reported at 15% [16]. Using the Self Reporting Questionnaire (SRQ) and probability sample of 510 households in two districts in the Kashmir Valley in 2005, De Jong et al. [7, 8] reported that psychological distress was experienced by 33% of their sample, with one-third reporting suicidal ideation. Research has also been conducted on the negative impact of natural disasters on mental health in the Kashmir Valley [17–19].

Although increased vulnerability to psychological distress in populations affected by protracted conflict is widely recognized [3], this often does not equate to increased access to care and treatment. The treatment gap for mental health is well-recognised, exacerbated in resource limited settings experiencing political instability where it is estimated to be in excess of 90% [20, 21]. The World Health Organization (WHO) has strongly advocated for the introduction of mental health in primary healthcare [22, 23] with research reporting successful implementation of primary care mental health programmes [24, 25]; however, few primary care workers know how to recognise an individual with mental health issues. In 1999, the government of India initiated the District Mental Health Plan (DMHP) with the intention of staggering a rolling out of community-based mental health services in all states of India [26]. The programme commenced in Jammu/Kashmir in 2004–2005, however, the 2012 National Mental Health Plan (NMHP), report results from a review of the DMHP stating it was barely functional in most districts [27] [28]. Médecins Sans Frontières (MSF) has provided mental health and psychosocial services in five districts of the Kashmir Valley since 2012.

Mental health is a socially constructed and socially defined concept; that is, different societies, groups, cultures, institutions and professions have very different ways of conceptualizing its nature and causes, determining what is mentally healthy, and deciding what interventions, if any, are appropriate [29]. Understanding that mental health is highly context-sensitive, influenced by cultural factors such as class, gender, ethnicity, religion, in addition to a range of social factors is imperative for the development of person-centered care and treatment.

We conducted a qualitative study as part of ongoing activities to better understand the burden of mental health distress in the Kashmir Valley and specifically explore community perceptions of and reactions towards mental illness, document current health seeking behaviours and identify perceived mental health service needs. This study was part of a larger multi-method study incorporating qualitative and quantitative methodologies as complimentary, although separate, study designs. Results of the quantitative arm, a large cross-sectional survey are presented elsewhere [11]. The findings from this study provide further insight into mental health needs in the Kashmir Valley, which will help increase relevance and impact of service delivery in Kashmir, whilst also serving as an advocacy tool for people-centred mental health programming and policy review.

## Methods

### Study setting

The Kashmir Valley is located in the landlocked state of Jammu and Kashmir in India with a total population of approximately 12.5 million [30]. Available mental health services in the Kashmir Valley follow a western biomedical model of care and treatment. Services are largely centralised in the main city of Srinagar. There is one dedicated psychiatric hospital, the Institute of Mental Health and Neurosciences (IMHANS), which provides inpatient and outpatient, care. Other major hospitals in Srinagar also offer psychiatric services, with a few psychiatrists operating private clinics.

### Study design and sample selection

A qualitative exploratory research design based on principals of grounded theory [31] was employed to ascertain community perceptions on mental illness, care seeking and service needs. We chose to include this qualitative arm to the cross-sectional prevalence study in recognition that qualitative methods enable further exploration of social phenomena that is pragmatic, interpretive and grounded in lived experiences.

Focus Group Discussions (FGDs) were conducted separately with groups of men and women in all ten districts of the Kashmir Valley between October–December 2015. One village was selected from each of the ten districts in the Kashmir Valley using a combination of convenience and snowball sampling. The FGD teams travelled with survey teams, collecting data for a large cross-sectional study, on each Tuesday during the ten week data collection period. Therefore, the village in which FGDs were conducted was dependent on where survey teams were travelling on the designated Tuesday. Adult participants (≥ 18 years of age) were identified using a combination of convenience and snowball sampling after initial contact with the village chief. The convenience sampling method was selected to obtain a group of individuals believed to be representative of the community, but chosen for ease of access [32]. In the first instance the village chief was asked to identify men and women ≥18 years of age to discuss ‘tension’ and ‘pareshani’ in their community. Clarity was provided that we were seeking a cross-section of the community with respect to age, and were enthusiastic to hear from individuals with and without a family member suffering from mental illness. In the second instance the individuals the village chief selected where then asked to identify others men/women in their community until we reached a sample size of 8–15 per group.

### Procedures

A semi-structured interview guide with open-ended questions, developed by TH & SA was used to promote discussion on the groups perceptions on the following areas; commonly recognized symptoms of psychological distress and mental illness, factors associated with having an impact on an individual’s mental health, community reactions to symptoms of psychological distress and mental illness, mental health help-seeking practices in the community, awareness of services available for the treatment and management of psychological distress and mental illness, the groups understanding of ‘counselling’ or ‘talk therapy’ and participants’ perspective on what services were required in order to better support mental health in their community (see Additional file 1 for the interview guide).

Trained Kashmiri bi-lingual male and female facilitators (AS&SA) and assistants facilitated separate group discussions with males and females, respectively. The assistants were responsible for notetaking and recording observations. The facilitators were Kashmiri clinical psychologists and the assistants were postgraduate sociology students from the Kashmir of University. The focus group team was pre-trained by the lead researcher, an experienced qualitative researcher (TH). Objectives of the study were explained to participants and verbal informed consent was obtained, including consent for audio recording. To ensure anonymity demographic data collected by the facilitator was restricted to age and gender. At the beginning of each FGD participants were reminded of the importance of maintaining confidentiality, that they had the right not to participate in any part of the conversation and that they were free to leave the group at any time during the discussion. No incentive was offered for participation. Focus groups were held in private rooms in a community building or private home nominated by the village chief, the average duration was approximately 70 min. Reflexivity [33], the process of reflecting on the researcher’s own knowledge and experience and how this may influence the research was considered throughout the research process. The research team conducted post-FGD debriefs on the journey back to Srinagar, where they discussed and reflected on interactions during the FGD, their impressions and the impact of the discussion on the researcher. Research diaries were also kept by each research team member as recommended by Lincoln and Guba [34].

Digitally recorded data were transcribed verbatim in Kashmiri and after regaining familiarity with the data, SA conducted the initial content analysis using ‘open’ coding [35]. Coding was cross-checked by TH, discrepancies were discussed, consensus reached and final codes entered directly into Microsoft® Office Excel. Continual referencing back to the data during analysis ensured that the analytical process was firmly grounded in the actual data [36, 37]. Once primary categories and relationships were developed, the transcripts were explored in depth in a second stage of coding [37]. During this stage connections between categories and relationships were identified and existing codes subcategorised and allocated to minor themes. This process is known in the literature as ‘axial coding’ or the practice of making connections [36, 37]. In this way more specific experiences concerned with perceptions of mental health/illness and access to service were given a title or name. The final stage of coding grouped minor themes into major themes, Strauss and Corbin (1990) describe this phase as the process by which all categories are structured around a central or ‘core’ category [31]. Transcripts were re-examined and quotes illustrating each of the identified themes selected. A sub-set of six randomly selected transcripts were translated into English, reviewed and coded using NVivo 11 software [38] by TH independently to assess validity and reliability of the joint-coding process. No major discrepancies were noted. Reflexivity in data analysis occurred through recognizing that the researchers influence the analysis through their own interpretations, underlying assumptions and unconscious biases. We attempted to minimize unconscious bias with the use of two analysts, one a clinical psychologist, and the other not a mental health professional. Reliability and transferability of findings was also confirmed through data saturation. Data saturation was reached within the first six focus group discussions, however for completeness and to ensure all ten districts were represented, focus group discussions were conducted in all 10 districts. The additional focus groups strengthened findings, although new codes and sub-themes emerged, no additional major themes were identified.

The study was approved by the Médecins Sans Frontières Ethics Review Board (ERB) (ID 1516), the Government Medical College Srinagar ERB (ID 19/ETH/GMC/ICMR), and the Australian National University Human Research Ethics Committee (ID 2015/516).

## Results

A total of 20 FGDs were conducted, one with females and one with males in each of the ten districts in the Kashmir Valley; 186 participants aged between 18 and 80 years of age attended. 95 men, median age 40 years, interquartile range (IQR): 27–48 years and 91 women, median age 40 years, IQR: 32–50 years. Results are presented under the main emergent themes. Panel 1 highlights the main themes with illustrative quotations.

### Recognised presentation of mental illness

The presentation of someone suffering from ‘*pareshani*’ or psychological distress was described in terms of physical, cognitive, social and behavioural characteristics. Commonly mentioned physical symptoms included; facial expressions reflecting sadness, hopelessness and ‘*tension*’, heart palpitations, blood pressure, ‘*ghabrahat*’ (Kashmiri term to describe nervousness), changed sleeping patterns, restlessness, weakness, crying always and weeping. Cognitive symptoms discussed included features of dissociation, mood fluctuations and instability, forgetfulness, inability to make decisions, ‘*physically there but mentally somewhere else*’, and ‘*in his own mind with his thoughts’*, excessive thinking, poor attention or concentration, lack of tolerance or patience, ‘*mind not functioning well’*. The social presentation of someone with psychological distress was described in terms of notable changes in social interaction, and changes in verbal communication. Symptoms discussed included social withdrawal and isolation, a change in speech – talking more or talking less than usual, speech content described as irrelevant or inappropriate, *Fanafilla* (the Kashmiri term to describe the lack of social boundaries and self-awareness that leads to culturally inappropriate behaviour, and sometimes used for someone who is spiritually connected to divine power) inability to listen to others, loss of interest in life (including in their family), and a lack of tolerance and patience with others. Behavioural presentations discussed related to expressions of aggression and substance use. Anger and aggression were discussed in all focus groups with references to both verbal and physical aggression. The inability to carry out tasks related to daily life and the act of roaming or aimless wandering were other commonly mentioned behavioural presentations.

Symptoms of psychological distress mentioned in the focus group discussions were cross-checked with symptoms listed in the HSCL-25 and HTQ-16. Commonly mentioned symptoms from focus group discussions present in one or more of the screening tools included nervousness, heart palpitations, restlessness, crying easily, sadness, hopelessness, thinking too much, loss of interest in things, changed sleeping patterns, forgetfulness, poor attention and concentration, inability to make decisions, aggression and anger, social withdrawal and isolation,

**Table:** 

Panel 1: Illustrative quotations from seven emerging themes on mental health in the Kashmir Valley	
**Presentation of psychological distress** su tchu har waqte mayues aasan taemis tchaeni aasan kah khushi bayei chu su alag thalag yecaan behuen beyi ne kaensi sith kath ne keh nea kah kaem ne yaecaan karun sirf dapaan bea baemha bea vucheha ne kaensi huend buuth nea bayee baemha aenigaetis be nea hasa vucheha gash te keheen ye chu aemis yaemis ye depression chu aasan. He is always sad, not happy at all, wants to sit alone, doesn’t talk, dislikes doing anything, doesn’t like to have anyone around him and wants to sit in darkness so that he will not see the light. All this happens to the person who is suffering from depression. 46-year-old female participant, Pulwama **Causative factors** Beyi gaye yeim haalat aezik yemaev haalaatav te oen vaariyaa keh vunkaes chuni haekaan insaan taeli aes ceer nacaan vacaan baaki cheez vunkaes chuni haekaan bache agar naebaer naeri, pareshaan che rozaan, paetii paetii chu paevaan aemis pakun khabar cha thaf ma yeyaes keh ma gachi baek cheez chu vunkaes chuni zindagi paeth barosae bae yoeri dravuus oeri yemaa vapas teith gayee zyadi yaeth saeni kasheeri manz haalat taem te oen vaariyaa dabaav. The present conditions [Socio-political conflict] have affected our life. There was a time when people used to enjoy at late hours. Today, if a child goes outside the house, parents remain tense/worried and look for him all the time as there is uncertainty and they think that he might get arrested or some other unlucky incident may happen to him. Due to uncertain conditions in Kashmir, a person does not know whether he will come back to home or not after going out of the house and this caused lot of stress/tension. . 40-year-old female participant, Pulwama **Community perceptions** ….yemis zaheni takleef aesi jinabali, temis tche aksar rozan insaan douryi Distance is maintained from the person who has mental illness. 55-year-old male participant, Srinagar ... Taemis bechaeris tcheni paesh yevaan theek tareeq keh... niche nazree tchis vuchaan ...That poor person is not treated well... Seen as inferior. 27-year- old female participant, Kulgam. ...mea basaan avoide tche karaan aemis yaemis ye zaheni takleef aasi ...I feel the person with the mental health problem is being avoided. 40-year-old female participant, Pulwama, **Help seeking practices** Pehlay peer kay pass laejatay hain aksar peer kay pass laejatay hain us kae baad doctor kay pass laejatay hain. First they are taken to a spiritual healer/peer, often taken to a peer and after that they are taken to the doctor. 27-year-old female participant, Baramulla	Tche na dapaan kah Doctor tchu tuethti, tensioni, yus tensionas wataan tchu, su tchu dawah wavah dewaan, ghacaan tche faraq, wini tchu tension tchu asaan su tchu oeruk yoeruk wanaan, temis tche wanan yi nimoen Doctoras Kum se kum gacaes faraq We say that there is a kind of ‘tensionee’ doctor who takes care of tension, he prescribes medicine and the person improves. When a person is in tension he can talk nonsense, then we tell them to take him to a doctor; at least he gets better. 45-year-old female participant, Srinagar **Barriers to accessing care and treatment** Jo loag gareeb hongay jis k pass koi guzaara nahin hoga vo aesay he chod daetay sonchtae hain ye apnay haal pae shayad mast hai tou rehnay dou hogaya theek tou khud he ho jaega nahin hogaya tou dekhaengay vo nahin karatay iska koi ilaj aesay b bohat loag hai.. People who are poor and cannot afford [treatment], leave such persons [persons with mental health problems] on their own, thinking that they will get better by themselves, or else we see, many people don’t even seek treatment [for such persons]. 18-year-old female participant, Baramulla Mental hospital hai lekin mental hospital pura throughout state me dou [1] he hai ya tou jammu me hai ya Srinagar me hai tou vahan pae ghareeb logun ka sources he nahi hai pohanchnae kay. There is the mental hospital but there [are] only two mental hospitals in the whole state. One is in Jammu and the other in Srinagar. To reach this place is not affordable. 60-year-old male participant, Anantnag **Perceptions of the effectiveness of treatment received** Eyess chhe wuchaan, bemaar chhe peeran nishh gachhan rozaan petmou paancxou, sheyou, ya dahou veryou petth. Dahou veryou patte chee tumann henzz mushkilaat badaan, tuman henzz haalat chhe waarya kharaab gachhan, patte chhe tuman ellaaj karun wariya mushkil gachhan What we see, the patients we observe they have been going to the faith healer from the last five years, six years or ten years. After ten years there problem has increased, there conditions worsens and it is very difficult to give them treatment. 20-year-old male participant, Bandipora Tum lukhh yumm aesse chhe ellaj karan chhenn paane zaana zehni sehatt kya gouv. Tuman Daktoran (khaas daktor) chhen pattah kya ellaj pazze eithenn bemaaran Karunn The doctors involved (specialist) do not know what treatment should be given to such patients.45-year-old-male participant, Kupwara **Perceived mental health service needs** Koi program hota har dusray teesray din yahan logun ko samjatay, Agar koi clinic he hota vo har ek ko maheenay me ek din kisi din jamah karkay kuch baat samjatay. There should be some programme every second or third day to make people aware. If there was a clinic here then it would gather and raise awareness among people. 27-year-old female participant, Anantnag.

### Perceived causative factors associated with psychological distress or ‘pareshani’

Perceived causative factors associated with poor mental health outcomes included physical and political insecurity, socio-cultural factors, socio-economic factors, physical, environmental and spiritual factors. Insecurity and events related to the ‘*Halaat*’ or the ‘situation’ of protracted conflict in the Kashmir Valley were discussed at length in all focus groups as a cause of poor mental health. Specific traumatic events were mentioned such as the death or disappearance of a loved one and exposure to cross firing, but it was also the ‘*feeling of insecurity’* that was discussed, ‘*it [the potential for insecurity] is always on our mind’*. Socio-cultural factors included those related to family conflict or family ‘*tension*’, stress associated with the inability to meet socio-cultural expectations such as marriage in early adulthood and the dowry system, the breakdown of socio-cultural norms, interpersonal conflict and for younger Kashmiri’s the pressures associated with familial and societal expectations related to academic performance. Interestingly, when suicide was mentioned in focus group discussions it was in the context of the pressures on young people to ‘*have good [academic] results’*. Unemployment was the most commonly discussed socio-economic factor perceived to have a causative association with poor mental health outcomes. Poverty, the cost of living, inability to provide for the family and lack of job security were also commonly discussed. Perceived physical causes of poor mental health related directly to illness, ‘*someone who has poor physical health will also have problem in the head – distress*’ or poor health in the family. In women’s focus groups infertility was discussed as a cause of psychological distress and poor mental health. Earthquakes and floods were the most discussed environmental causes of poor mental health, both of which lead to loss of loved ones, loss of property and feelings of ‘*terror*’ and ‘*fear*’. The physical isolation that occurs during the winter season due to heavy snowfall was also perceived to be a cause of psychological distress. Spiritual causes of psychological distress were predominantly attributed to a djinn, ‘*tasruf*’ (possession) or fairies (pari). Other spiritual causes that were discussed included ‘*Nazar*’ (the evil eye), a curse put on one person by another. An ‘act of God’ was also indicated as a possible source of mental illness; ‘*this can be from the Almighty’*.

### Community perceptions of mental illness

When discussing how members of the community perceive an individual with symptoms of mental illness emphasis was placed on the stigma associated with mental illness. This included stigma associated with symptoms, treatment seeking and more broadly societal stigma. When someone displays recognisable symptoms of mental illness the stigma associated with the symptoms is evident in the reported response of members of the community towards these people; ‘*everyone does not treat them well*’, ‘*we don’t behave well with them’*, ‘*some ignore them, don’t think about them, avoid them’*, ‘*some taunt, tease them, make fun of them*’. Stigma associated with treatment seeking was associated with community perceptions that someone is ‘*paagal*’ (crazy), ‘if we take them to a tension doctor people will think they are mad and call them ‘*paagal*’, ‘*people are afraid of going or taking a person to the mental hospital because it is still called as mental asylum/hospital’*. The long term impact of societal stigma was discussed with respect to young people, who have had a history of mental illness, being unable to marry, ‘*we should hide it from others so that others cannot get to know this if the person is of marriageable age*’, ‘*even if he gets well, no one will marry him or he will not be given a job in a factory or company*.’

### Help seeking practices for the management of symptoms of mental illness

Participants in all focus groups discussed mental health help seeking behaviour in terms of a combination of socio-cultural and biomedical services. Traditional healers include ‘*peer saab’* (spiritual healer), are often preferred to biomedical services as they are accessible (usually in the same village or nearby), and share cultural knowledge, beliefs and practices. The *peer saab* uses amulets *‘taweez*’, verses from the Koran, and instructs the individual to perform ‘*niyaaz*’/*‘khatam-e-shaif’* ‘(ritual acts that aim to cure them). If treatment is unsuccessful with the peer the family may seek out another *peer saab* or go to a hospital to see a doctor. Biomedical treatment was referred to by all in terms of medication. It is common for people to adhere to both treatment prescribed from a medical doctor and from the *peer saab*, this has been given the colloquial term of ‘*Dua Ti Dawa Ti’*, both prayer and medicine. Pharmacists, locally referred to as a ‘*compounder*’ were also discussed as a source of treatment for symptoms of psychological distress. Taking a patient to the psychiatric hospital in Srinagar (the only psychiatric hospital in the Kashmir Valley), referred to by participants as the ‘mental hospital’, was viewed as a last resort, a decision a family takes when all other resources and options have been exhausted.

#### Perceptions of the effectiveness of treatment received

Mixed perceptions with respect to the effectiveness of treatment received were commonly discussed. The necessity for multiple investigations and tests in the biomedical system was discussed along with divergent views on the effectiveness of treatment; ‘*we have taken her to every doctor and she is not getting improved*’, ‘*the doctor gave me medicine but I was not able to tolerate it*’, ‘*tension doctors give medicine and the patient reports that he gets improved*’. The common practice in the biomedical system of subjecting the symptomatic individual to multiple investigations was a predominant theme; ‘*[we take them to] multiple specialists and undertake many investigations, [many] rupees are spent on specialists and investigations’*, ‘*too many investigations and tests…symptoms get worse until he is finally taken to the mental hospital’*. The effectiveness of treatment was at times described in terms of ‘fate’, ‘*if it is in his fate he will get better’*. The effectiveness of treatment received by traditional healers was also met with divergent views; ‘*these patients can’t get better by treatment from peer*’, ‘*they have told me to take her to the shrine, there she will get treated’*.

#### Barriers to accessing care and treatment

Perceived barriers to accessing care and treatment included financial constraints, distance to services, lack of physical infrastructure such as roads and transport, and lack of knowledge of available services. Financial constraints to accessing services was viewed as a common barrier, ‘*if a person does not have money in the pocket then what will he do*. Distance to services had an impact on the type of care accessed; ‘*the peer is nearby and less costly*’, ‘*we don’t have a doctor or hospital…we get treatment from the compounder [pharmacist]*’, ‘*there is no service for people with tension to get first aid*’. Inadequate physical infrastructure was mentioned in terms of the need for adequate roads and transport; ‘*to find [a] doctor we need transport*’, ‘*we don’t have roads’*. Lack of knowledge of available mental health services was emphasised in all focus group discussions; ‘*we have no knowledge of services’, ‘we don’t have any guidance on this, we only take them to a doctor’*. Knowledge was restricted to faith healers, medical doctors and the mental hospital, ‘*pagalkhana’, ‘we have heard there is a dimagka (brain/mind) doctor in Srinagar’*. In response to the question on their understanding of ‘counselling’ or ‘talk therapy’ some participants had little to say; ‘*we don’t know about this’*, ‘*we have not heard about this’*. Others demonstrated a perception of the concept, described counselling as ‘*advice*’, ‘*giving sympathy*’, ‘*treatment*’, ‘*physical relaxation exercises*’, ‘*a program on the radio*’, ‘*brainwash that you don’t have a problem*’, ‘*making people aware of their problem and how to manage them*’, ‘*this can be a responsible wise person in the community who can advise*’.

### Perceived mental health service needs

#### Health service needs

Participants identified a number of services as necessary for the management of mental health in their communities including needs related to health services, physical infrastructure, socio-economics and skill development and community awareness programs. Emphasis was placed on the need for greater proximity to mental health services; ‘*whatever is needed for this [mental illness] should be here, it [mental health services] should be in every district*’, ‘*we should have some kind of clinic here for this [mental illness]*, *provide them a safe space so that they can vent their feelings and have someone to listen to them empathetically.* Few participants had knowledge of specialist mental health service providers, beyond the ‘mental hospital’. Those who had said that this service was not readily available to them or their community.

Importance was placed on training health care workers and doctors to be able to recognize and manage symptoms of psychological distress and mental illness; *‘[a need for] staff who can understand and motivate such [mentally ill] people’*, ‘*[a need for] staff that understand others museebath (troubles)*’, ‘*[the community needs] Dilbari karin – people who talk in such a way that they [those with mental illness] come out of this’*. Physical infrastructure to improve access was commonly referred to as both a barrier to accessing services and as a service need, ‘*we should have a road*’. Socio-economic pressures were recognized as having a negative impact on mental health and as such employment, business development, skill development and employment for women were all mentioned as service needs which would improve overall mental health in the community; ‘*there should be some way to earn a livelihood’*, *‘[we need] some kind of employment for women’*, ‘*some business needs to be started here’*, ‘*[we need] a centre for learning some kind of work and keeping busy’*.

A cross-cutting theme under perceptions of community mental health service needs was the need for community awareness programmes. The significance of mental health issues in communities came across strongly in the focus groups with all groups expressing that this was a very important issue in their community, however they felt unequipped to manage people with symptoms of psychological illness. Participants expressed a desire to know how to support members of their family and community with signs and symptoms of psychological distress. ‘*there should be some programme then people could understand*’, ‘*we should have a facility to show us how to do some kind of first aid [for mental illness]*.’

## Discussion

We report the findings of the qualitative arm of a larger research study exploring mental health in the Kashmir Valley, India [39]. The aim of this qualitative study was to gain insight into community perceptions on psychological distress, care seeking and service needs. To our knowledge, this is the first study to combine a cross-sectional population based survey with a qualitative exploration of community perceptions on psychological distress in a population affected by protracted conflict. The findings from the qualitative exploration, reported here, add meaning to the epidemiological evidence and serve to inform programmatic and policy decisions. The cross-sectional survey that occurred concurrently with this qualitative inquiry demonstrated high levels of psychological distress in the Kashmiri population, alongside multiple traumatogenic experiences [11]. Our qualitative inquiry served to further validate the selection of the psychometric screening tools for our cross sectional survey and provided insight into the lived experience of the manifestation of psychological distress in the population. Our findings demonstrate the importance of acknowledging universal idioms of psychological distress in addition to culturally defined expressions and understandings of psychological distress.

While it is well recognized that the development of contextually valid psychometric instruments is the ideal in order to capture local expressions of distress [40], we found common locally recognized symptoms of psychological distress to be synonymous with symptoms listed in the Hopkins Symptoms Checklist (HSCL-25) and the Harvard Trauma Questionnaire Posttraumatic Stress Disorder checklist (HTQ-16). Thus providing further validation of the use of these instruments in the cross-sectional survey and for potential auxiliary use in the community as tools for early detection and referral [10, 11]. In saying this, we recognize that there is a need for a Kashmiri instrument that is grounded in and captures additional local expressions of distress. An approach to developing such an instrument would involve extensive ethnographic research, content validation and evaluation of psychometric properties. Also, one might question the resulting conceptual comparability of such a new instrument. Until such time as an indigenous instrument is developed the culturally adapted and validated HSCL-25 and HTQ-16 [10] have demonstrated a ‘good-fit’ for use in the Kashmiri population.

The practice of seeking treatment from traditional/spiritual healers for signs and symptoms of psychological distress is commonplace. Kashmiri’s adopt an explanatory model of psychological distress in part defined by the spiritual and/or traditional influences grounded in Islamic religion/faith. The biopsychosocial model is one of the dominant treatment approaches in psychiatry and religious and spiritual orientation/beliefs are considered as an important psychosocial factor in human life. In 1984, the World Health Assembly addressed the importance of a spiritual component in health [41], and one of the founding fathers of modern psychiatry, Carl Jung, emphasized the importance of religion in attaining the psychological health and enjoying a normal state of mental well-being [42]. In the Kashmir Valley, it is common practice for individuals to discuss with Imams and religious leaders their difficulty in dealing with social and psychological problems; people will often consult with religious or faith healers well before they seek western medical services. Positive expectations of the effectiveness of the ‘treatment’ received by traditional/spiritual healers, such as the use of amulets, verses from the Koran and instructions to perform ritual acts known as ‘*niyaaz*’ or ‘*taweez*’, gave meaning to psychological symptoms. However, a difference in expectations of effectiveness of treatment and the lived experience were expressed and further explored with the favoured treatment seeking being *Dua Ti Dawa Ti* (prayer and medicine). The role of the religious leader is to provide advice which would be in relation with the Islamic principles. Beliefs play an important role in terms of managing and treating mental health issues as shown through various studies [43–45]. Traditional/spiritual healers are recognized as a valuable component of the health system and therefore need to be educated about mental health issues, its management and treatment. Kashmiri’s are likely to continue to seek care and treatment from traditional healers alongside that of medical practitioners. Awareness among these leaders regarding mental health, and signs of psychological distress will facilitate early detection and intervention, as they are often the first point of contact and more easily accessible in the community.

The commonality in which poor treatment outcomes were reported by participants in the focus groups may be explained by the lack of recognition of somatisation by general health service providers, or the tendency to express psychological distress as somatic symptoms. Somatisation is well recognized by Kashmiri psychiatrists [46, 47] and in other conflict affected populations [48, 49], this poses a challenge for early diagnosis and referral if health service providers are not adequately trained to recognize somatic symptoms, leading to treatment delay and poor prognosis [46]. A recognized challenge in Kashmir is that general health professionals have little or no understanding of somatization as an indicator of psychopathology, and so suffering individuals are referred to neurologists, cardiologists and other specialized medical services where they undergo a battery of tests, resulting in a huge cost burden to the family [50]. The help seeking back-and-forth behaviour between pursuing care from traditional/spiritual leaders and the medical system with perceived little or no reported improvement was shown in this study to result in increased frustration and increased financial cost to the patient. This is not unique to Kashmir. A systematic review by Murray et al. [51] reported that misdiagnosis of somatic symptoms can lead to increased monetary costs to the patient and the health system with the risk of exacerbation of symptoms and higher impairment. There is a need to improve the mental health literacy among health professionals, traditional/spiritual healers and communities in order to facilitate early detection of psychopathology and psychological distress and ensure timely access to care and treatment. There is also a need for research that tests the effectiveness of interventions and builds a basis for evidence based practice in the Kashmiri context, with a focus on early detection, management and appropriate referral.

The World Health Organization (WHO) has strongly advocated for the introduction of mental health in primary health care [52, 53]. In 1999 the government of India initiated the District Mental Health Plan (DMHP) with the intention of staggering a rolling out of community based mental health services in all states of India [28]. The program commenced in Jammu/Kashmir in 2004–2005, however, the 2012 National Mental Health Plan (NMHP), reports results from a review of the DMHP, stating it was barely functional in most districts [28]. The 2012 NMHP suggests a renewed commitment by the government of India to address the mental health needs of its population and called for research which could ‘offer insights as well as pathways for change’. [28] However, challenges continue to plague the effective roll out of the NMHP across India with leadership, governance/administration, financial and human resources, community and stakeholder participation, training of health staff including community mental health professionals, and robust mechanisms for monitoring and evaluation all being implicated as determinants for successful implementation [54]. Other studies have shown community engagement, mobilization and sensitization have been recognized as key components in ensuring successful implementation of mental health programming [44, 55], however, community based mental health programmes have been implemented with mixed success [56–58]. A recognized challenge is the recruitment, training and retaining mental health service providers [59, 60]. Commitment at all levels in the health system are needed to address the mental health needs of local populations.

Trusting and supportive relationships between service providers and recipients [44, 61],group-based programs [55, 62] and culturally relevant activities [44, 60, 61], have been shown to increase social connectedness, decreasing isolation and thereby improve resilience. However, the evidence-base is weak at best.^63^ What is lacking in the design and implementation of mental health programs in conflict affected populations is a comprehensive approach co-designed, implemented and evaluated with and by communities.

The main strength of this study is the participation of nearly two hundred individuals, representing a cross-section of ages across different regions of the Kashmir Valley. The collective experience of protracted conflict provided a common experience with participants in the focus group discussions expressing a shared and individual testimony; related issues associated with psychological distress were discussed with seemingly no/little reservation in spite of the recognised social stigma attached to symptoms of psychological distress. Focus groups are recognised as an effective means of gaining a ‘collective testimony’ of issues of importance to communities, drawing out a narrative and capturing how meanings around shared experiences are co-constructed. A limitation associated with focus group discussions is that participants may perceive varying levels of freedom to share experiences and views, particularly if they are known to differ from popular views. This may lead to the introduction of social desirability bias with some participants suppressing views or self-censoring what they disclose and the repetition of more acceptable ‘community narratives’. The facilitators, in this study, worked to reduce the impact of social desirability bias and dominant responder bias by encouraging the expression of different opinions and views, and providing opportunity for all participants to contribute to the discussion. The use of convenience and snow ball sampling methods may have introduced bias in that participants are not representative of the entire population and likely do not include the voice of marginalized populations. Non-probability sampling techniques have been recognized as viable alternatives in exploratory research with constraints on time and resources.^64^ This method of sampling was considered acceptable for our study aim and design. The use of Kashmiri clinical psychologists as facilitators during the focus group discussions was viewed as a strength, their in-depth knowledge of the manifestation of mental distress within their own population and the language used to describe idioms of distress ensured continuity of discussions. Their skill in facilitating discussions and probing for further information/clarification were also viewed as an asset. However, it must be acknowledged that prior knowledge and experience may also lead one to make assumptions or unconsciously direct the research data collection/analysis and interpretation. The impact of researcher induced bias was minimized through open discussions and reflections by the team throughout the research process, listening to recorded sessions and providing feedback, keeping research diaries and the cross-checking of thematic analysis by an experienced qualitative researcher who did not have a background in psychology.

## Conclusion

Our research has highlighted a need for co-constructed models of community based mental health service delivery, where communities, medical professionals, religious leaders and traditional/spiritual healers are engaged in the design, implementation and evaluation of service delivery. Knowledge and understanding of local conceptualisations in tandem with universal idioms of distress is essential to inform public mental health program and policy

## Supplementary information

**Additional file 1: ** Interview Guide for Focus Group Discussions

## Data Availability

Access to data can be provided on reasonable request to and approval from the Medical Director at Médecins Sans Frontières, Amsterdam the Netherlands.

## Abbreviations

AFSPA: Armed Forces Special Powers Act
ERB: Ethics Review Board
FGD: Focus group Discussion
HSCL-25: Hopkins Symptoms Checklist, 25 items
HTQ: Harvard Trauma Questionnaire
IMHANS: Institute of Mental Health and Neurosciences
MSF: Médecins Sans Frontières
OHCHR: Office of the United Nations High Commission for Human Rights
PSA: Public Safety Act
PTSD: Posttraumatic Stress Disorder
