# Supplementary material for: Dua Ti Dawa Ti: understanding psychological distress in the ten districts of the Kashmir Valley and community mental health service needs
Source: Confl Health. 2019 Dec 12;13:59. doi: 10.1186/s13031-019-0243-8 (PMC6909639; doi:10.1186/s13031-019-0243-8)
Supplement: Supplementary file 1 — Additional file 1: Interview Guide for Focus Group Discussions [file 13031_2019_243_MOESM1_ESM.docx]

**Supplementary Appendix**

**Interview Guide for Focus Group Discussions**

1. When you hear the phrase 'mental health' what sorts of things come to your mind?

**Yaeli tueh lafz “zaheni Sehat” cheiv bozaan toehi kya chu taemi veezi zehnas/ dimagas manz yevaan?**

1. When you hear the phrase 'mental health problems’ what sorts of things come to your mind?

**Yaeli tueh lafz “zaheniTakleef” cheiv bozaan toehi kya chu taemi veezi zahnas/ dimagas manz yevaan?**

**Probe to Q2:** How does a person with tension or pareshani look to you? What makes you think they are suffering from tension or pareshani?

**Yaemis insanas Pareshani/ Tension ya zaheni takleef chu aasan su kueth chu basaan? Toehi kith kaen basaan temaen cha pareshani ya tension?**

1. Could you tell me about the factors that you think relate to/ have impact on 'mental health problems'. ?

**Toehi kya basaan koem vajuhaat hakaen aasith yemaev sith Zaheni Takllef hakaen gacith?**

1. How does your community react to people with mental health problems?

**Tuhund maashri kithpaet chu paesh yevaan temaen lukan sith yemaen Zaheni Takleef aasi?**

1. Who do people in your community go to for care and treatment when they, or one of their family members show signs of having a mental health problems?

**Agar tuhendis maashiras ya gharas manz kaensi Zaheni Takleef aasi su koeth chu gacaan taem ki ilaaji khaetri/ baabat?**

1. What services are you aware of for people with mental health problems?

**Zaheni Takleefan manz mubtilaa lukan khaetri komaen khidmatan/sahulataen heinz zankaari chaev toehi?**

1. What are the perceptions of people in your community if you or one of your family members seeks help from Mental Health services?

**Tuhendis mashiras manz kueth nazariyaa chu lukan huend aath mutlik ke agar kah zaheni takleef khaetri kuni khadimat/ sahuliyataen huend madat neyee?**

1. What do you think your community needs to help improve the mental health of the people?

**Toehi kya chu basaan Zaheni Sehat behtar banavni khaetri tuhendis/saenis maashiras komaen cheezan hinz zarurat chi?**

1. What comes to your mind when you think about counselling or therapy?

**Tuhendis zehnas manz kya chu yevaan yaeli tueh “Counselling” ya “Psychotherapy” mutlik cheiv sonchaan?**

**Exit question**

1. Is there anything else you would like to say about what services your community need in order to better support people with mental health problems?

**Toehi cha basaan beyi keh vaninich che zarurat yaemi sith saenis maashiras manz temaen lukan madat vaati yemaen Zaheni Takleef chu?**
